# Acute exposure to air pollutants increase the risk of acute glaucoma

**DOI:** 10.1186/s12889-022-14078-9

**Published:** 2022-09-20

**Authors:** Liping Li, Yixiang Zhu, Binze Han, Renjie Chen, Xiaofei Man, Xinghuai Sun, Haidong Kan, Yuan Lei

**Affiliations:** 1 Department of Ophthalmology & Visual Science, Eye Institute, Eye & ENT Hospital, Shanghai Medical College, Fudan University, Shanghai, 200031 China; 2 NHC Key Laboratory of Myopia, Chinese Academy of Medical Sciences, and Shanghai Key Laboratory of Visual Impairment and Restoration, Fudan University, Shanghai, 200031 China; 3 School of Public Health, Key Lab of Public Health Safety of the Ministry of Education, NHC Key Lab of Health Technology Assessment, IRDR ICoE on Risk Interconnectivity and Governance on Weather/Climate Extremes Impact and Public Health, Fudan University, P.O. Box 249, 130 Dong-An Road, Shanghai, 200032 China; 4 Shanghai Typhoon Institute/CMA, Shanghai Key Laboratory of Meteorology and Health, Shanghai, 200030 China; 5 Department of Ophthalmology, Xinhua Hospital Affiliated to Shanghai Jiao Tong University School of Medicine, Shanghai, China; 6 Children’s Hospital of Fudan University, National Center for Children’s Health, Shanghai, China; 7 State Key Laboratory of Medical Neurobiology and MOE Frontiers Center for Brain Science, Institutes of Brain Science, Fudan University, Shanghai, 200032 China

**Keywords:** Air pollutants, Acute glaucoma, Case-crossover study

## Abstract

**Background:**

Ambient air pollution is related to the onset and progression of ocular disease. However, the effect of air pollutants on the acute glaucoma remains unclear.

**Objective:**

To investigate the effect of air pollutants on the incidence of acute glaucoma (acute angle closure glaucoma and glaucomatocyclitic crisis) among adults.

**Methods:**

We conducted a time-stratified case-crossover study based on the data of glaucoma outpatients from January, 2015 to Dec, 2021 in Shanghai, China. A conditional logistic regression model combined with a polynomial distributed lag model was applied for the statistical analysis. Each case serves as its own referent by comparing exposures on the day of the outpatient visit to the exposures on the other 3–4 control days on the same week, month and year. To fully capture the delayed effect of air pollution, we used a maximum lag of 7 days in main model.

**Results:**

A total of 14,385 acute glaucoma outpatients were included in this study. We found exposure to PM_2.5_, PM_10_, nitrogen dioxide (NO_2_) and carbon monoxide (CO) significantly increased the odds of outpatient visit for acute glaucoma. Wherein the odds of acute glaucoma related to PM_2.5_ and NO_2_ were higher and more sustained, with OR of 1.07 (95%CI: 1.03–1.11) and 1.12 (95% CI: 1.08–1.17) for an IQR increase over lag 0–3 days, than PM_10_ and CO over lag 0–1 days (OR:1.03; 95% CI: 1.01–1.05; OR: 1.04; 95% CI: 1.01–1.07).

**Conclusions:**

This case-crossover study provided first-hand evidence that air pollutants, especially PM_2.5_ and NO_2_, significantly increased risk of acute glaucoma.

**Supplementary Information:**

The online version contains supplementary material available at 10.1186/s12889-022-14078-9.

## Introduction

The onset and progression of multiple diseases connected closely with ambient air pollution [1] including cardiovascular disease [2–6], type 2 diabetes mellitus [7–10], chronic obstructive pulmonary disease [11, 12], and even cancer [13, 14] and mortality [15]. Recently, the association between glaucoma and ambient air pollution is emerging [16, 17].

Glaucoma is the leading cause of irreversible blindness in the world, which is estimated more than 70 million persons aged 40–80 suffering from this condition worldwide [18, 19]. According to the risk factors, etiology, duration, symptoms, treatment, and prognosis, glaucoma is classified into different types [20]. Both angle closure glaucoma and glaucomatocyclitic crisis can have acute onset. Angle closure glaucoma is presented with an anatomically closed angle which was casued by apposition of the iris [21]. A closed angle prevents the outflow of aqueous humor and hence causes elevated intraocular pressure (IOP) [22]. In acute primary angle closure attack, IOP could reach to 30 mmHg or even higher. There are several risk factors related to angle closure such as female, older age, and Asian ethnicity (e.g. Chinese) [23] The clinical data from our hospital reveals that primary angle closure glaucoma accounted for 50–55% glaucoma patients [21]. Glaucomatocyclitic crisis (also called Posner-Schlossman syndrome), uaually involves recurrent episodes of increased IOP, acute anterior chamber inflammation and keratic precipitates [24]. The etiology of glaucomatocyclitic crisisris is not very clear which may be involved of virus infections. It has the similar clinical manifestation like an acute angle-closure glaucoma because of the initial sudden and remarkable IOP elevation and the mild anterior chamber inflammatory.

High IOP has a similar pathological mechanism with high blood pressure [25]. The association of ambient air pollution with hypertension and blood pressure was investigated by numerous studies [26–29]. A meta-analysis, which searched seven international and Chinese databases, showed significant associations of long-term or short-term exposures to ambient air pollution with blood pressure and hypertension [30]. In recent years, particulate matter pollution was related to the incidence of self-reported glaucoma or unclassified glaucoma according to the epidemiological studies [31–34]. And our previous studies showed mice exposed to ambient air pollutants lead to ocular hypertension [35, 36]. However, each type of glaucoma has its own distinctive etiology, it is important to know which types of glaucoma patients are affected by air pollution so that appropriate cautions can be made.

The purpose of the current study is to investigate the impact of air pollutants on the incidence of acute glaucoma attacks including acute angle closure glaucoma and glaucomatocyclitic crisisris. The analysis is conducted based on the outpatient data from two hospitals in Shanghai. Air pollutants include PM_2.5_ (particulate matter ≤2.5 μm in aerodynamic diameter), PM_10_ (particulate matter with an aerodynamic diameter < 10 μm), sulfur dioxide (SO_2_), nitrogen dioxide (NO_2_), carbon monoxide (CO) and ozone (O_3_).

## Methods

### Design and population

**Fig. 1:**
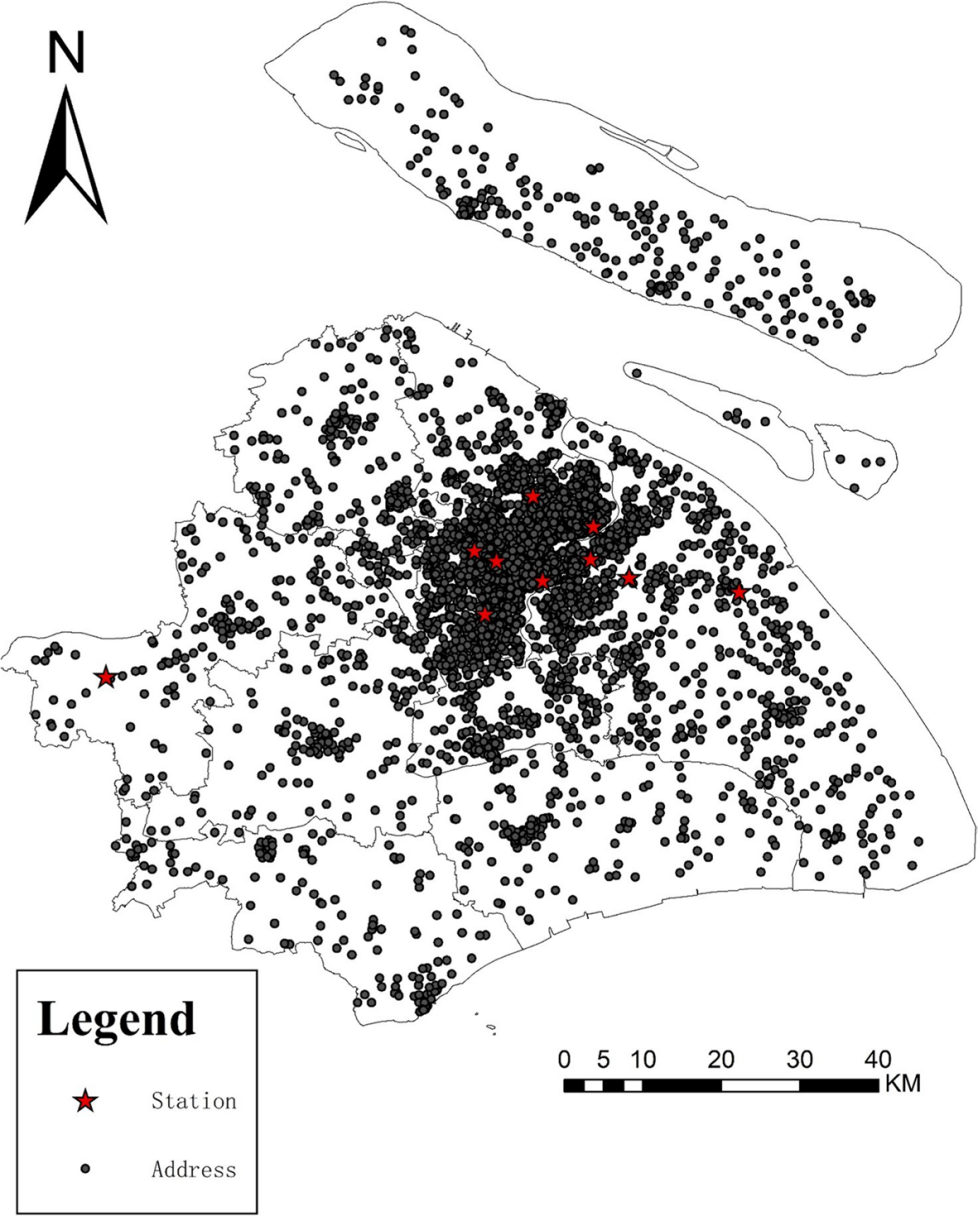
Address of patients for acute glaucoma attack and air quality monitoring stations in Shanghai, China, during 2015–2021

Date on acute glaucoma outpatient visits were collected between 1, January, 2015 and 31, Dec, 2021 from the Eye Ear Nose and Throat Hospital of Fudan University and Xinhua Hospital Affiliated to Shanghai Jiao Tong University School of Medicine in Shanghai, China. The inclusion and exclusion procedures were shown in Fig. S1. All patients clinically diagnosed with acute angle closure glaucoma or glaucomatocyclitic crisisris by physicians were regarded as acute glaucoma attack and were included in this study. Demographic characteristics, including age, gender, residential addresses and date of outpatient visits were collected. Pearson correlation analysis was conducted to examine the correlations between the air pollutants and meteorological variables. The patients without demographic information, aging under 18 or above 85 years old, and living out of Shanghai were excluded. Moreover, the patients with glaucoma surgery history, prescribing for medicines, suspected as glaucoma were excluded as well. Totally 14,385 cases living in Shanghai city were incorporated into this study (Fig. 1). The study protocol was approved by the Institutional Review Board (IRB) of the Eye Ear Nose and Throat Hospital of Fudan University (IRB#2022027) and adhered to the tenets of the Declaration of Helsinki. ﻿The informed consent was waived by the Institutional Review Board (IRB) of the Eye Ear Nose and Throat Hospital of Fudan University.

A time-stratified case-crossover design was applied to evaluate the potential associations of air pollution exposure and outpatient visits for acute glaucoma. In this design, each subject serves as his or her own control by selecting 3–4 control days matched to other days on the week of the same month-year of the outpatient visit day. This design could provide unbiased effect estimate and control the long-term trend and seasonal pattern [37, 38].

### Exposure assessment

Air pollution data was derived from the nearest air quality monitoring stations to participants’ address on China’s National Urban Air Quality Real-time Publishing Platform. We included the data of the daily (24 h) levels of PM_2.5_, PM_10_, SO_2_, NO_2_, and CO and daily 8 h maximum averages O_3_ in the analysis. Daily meteorological data (average temperature and relative humid) recorded from the nearest weather stations were also acquired in the China Meteorological Data Sharing Service System (http://data.cma.cn/).

### Statistical analysis

A conditional logistic regression model with polynomial distributed lag model (PDLM) was conducted to quantitatively examine the association between air pollution with outpatient visits for glaucoma. The results were presented as the odds ratios (ORs) of glaucoma incidence associated an interquartile range (IQR) with the 95% confidence intervals (CI) of the air pollutants. ﻿The PDLM was widely applied to estimate the lagged impact of environmental factors on health. As flexible “cross-basis” functions, air pollution indices was defined as combinations of natural cubic spline with 2 degrees of freedom (df) for exposure space and 3 df for lag space [39, 40].

To fully capture the delayed effect of air pollution, we used a maximum lag of 7 days in PDLM. Furthermore, considering the nonlinear confounding effects of weather conditions, the model included a smoothing function using natural splines with 6 df for the 3- day moving average temperature and 3 df for 3-day moving average relative humidity to adjust for the nonlinear confounding effects of weather conditions. The public holidays were also adjusted in the model. Furthermore, we used conditional logistic regression model combined with distributed non-linear models (DLNM) to describe the exposure-response associations of air pollution with risk of glaucoma. By examining and plotting cumulative effects, lag days with significant effects was found and then applied to plot the exposure-response (E-R) association. The models fit from the 0.1th to 99.9th percentiles of the concentrations of each pollutant, respectively. We also performed subgroup analyses by gender (male and female) and age (18–44 and 45–85 years) to assess the modifying effects of demographic features. We tested the statistical significance of differences between effect modifications by calculating the 95% confidence interval as

$$\hat{\Big({\mathrm{Q}}_1}-\hat{{\mathrm{Q}}_2}\Big)\pm 1.96\sqrt{\hat{{{\mathrm{SE}}_1}^2}+\hat{{{\mathrm{SE}}_2}^2}}$$

where $\hat{{\mathrm{Q}}_1}$ and $\hat{{\mathrm{Q}}_2}$ represent the estimates for the 2 categories, and $\hat{{\mathrm{SE}}_1}$ and $\hat{{\mathrm{SE}}_2}$ represent their corresponding standard errors, respectively [41]. To address the multiple testing problem, we applied the Bonferroni correction to adjust the significance the threshold. In addition to the main model described above, we fitted two-pollutant models, each of which included adjustment for one of the other five pollutants in the sensitive analysis.

All statistics analysis were conducted with R software (Version 4.0.2, R Foundation for Statistical Computing, Vienna, Austria). We used the “survival” and “dlnm” packages to fit the conditional logistic regression model and DLNM, respectively.

## Results

### Descriptive data

Finally, a total of 14,385 medical records of glaucoma outpatients in Shanghai, China, from January 2015 to Dec 2021 were finally included. Wherein 40.9% (5887) were male and the average age was 56.79 (±15.33) years old. Geographic distribution of the included participants were shown in Fig. 1.

Statistics on air pollution levels and weather conditions on outpatient visits day throughout the study period were summerized in Table 1. During the study period, the mean ( ± standard deviation, SD) 24-hour level of PM_2.5_ and PM_10_ were 32.3 (±21.3) μg/m^3^ and 47.3 (±29.5) μg/m^3^, which were higher than the ﻿recommended ambient air quality standard by ﻿World Health Organization (WHO) for gaseous air pollution. The daily SO_2_, NO_2_, CO and O_3_ exposure on the outpatient day were 7.0 (±3.8) μg/m^3^, 40.5 (±19.3) μg/m^3^, 0.7 (±0.3) mg/m^3^ and 92.3 (±43.4) μg/m^3^, respectively. The IQR values of PM_2.5_, PM_10_, SO_2_, NO_2_, CO and O_3_ were 26.0 μg/m^3^, 35.0 μg/m^3^, 5.0 μg/m^3^, 27.0 μg/m^3^, 0.5 mg/m^3^, 62.0 μg/m^3^, respectively. For meteorological features, the mean (±standard deviation) of temperature and relative humid were 18.9 (±8.3)°C and 75.9 (±13.6)%. In Table S1, the correlation of air pollutants indicated a strong statistical significance (*P* < 0.01) with the strongest correlation being between PM_2.5_ and PM_10_ (r value is 0.672), and then between PM_2.5_ and CO (r value is 0.671).

**Table 1:** Summary statistics on air pollution and meteorological exposure on outpatient day for acute glaucoma attack throughout the study period

Variables	Mean ± standard deviation	Min	25th percentile	50th percentile	75th percentile	Max
SO_2_ (μg/m^3^)	7.0 ± 3.8	1	4.7	6.2	8.4	60.2
NO_2_ (μg/m^3^)	40.5 ± 19.3	1.7	26.8	36.8	50.3	158.4
CO (mg/m^3^)	0.7 ± 0.3	0.1	0.5	0.7	0.8	2.3
O_3_ (μg/m^3^)	92.3 ± 43.4	1.4	60.9	86.1	116.8	304.8
PM_10_ (μg/m^3^)	47.3 ± 29.5	5.5	29.4	40	58	494.3
PM_2.5_ (μg/m^3^)	32.3 ± 21.3	2.6	17.1	26.4	41.2	107.7
Temperature (°C)	18.4 ± 8.3	−4.7	10.9	18.9	25.6	35.3
Humidity (%)	75.4 ± 13.6	32.5	66.4	75.9	85.5	100

*Abbreviations*: *PM*_2.5_ particulate matter with an aerodynamic diameter less than 2.5 μm, *PM*_10_ particulate matter with an aerodynamic diameter < 10 μm, *SO*_2_ sulfur dioxide; *NO*_2_ nitrogen dioxide, *CO* carbon monoxide; *O*_3_ ozone

### Regression results

**Fig. 2:**
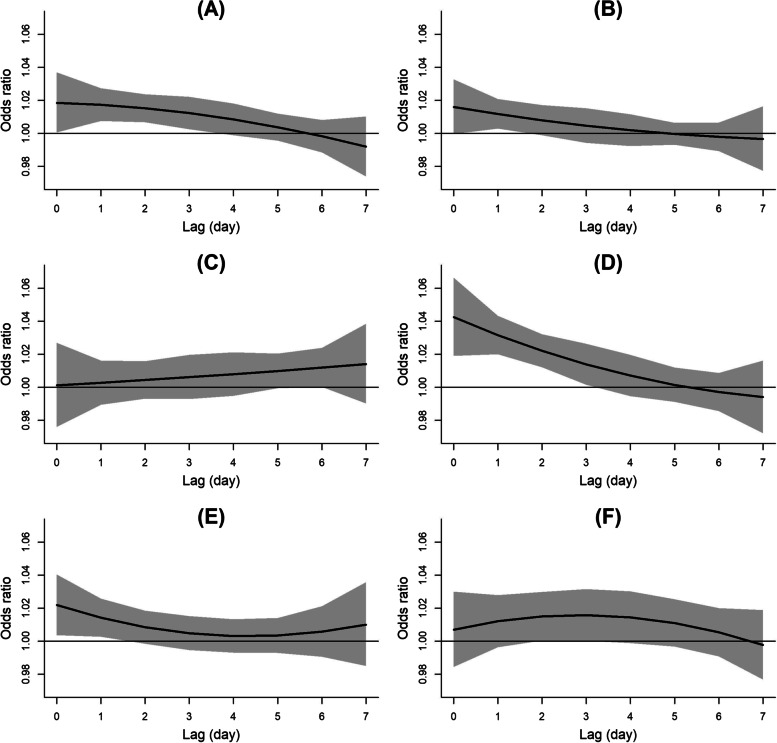
Overall lag structure in association of ambient air pollution exposure with acute glaucoma attack on different lag day. Panels A to F were the associations between OR of acute glaucoma attack and air pollution exposure, including (**A**) PM_2.5_, (**B**) PM_10_, (**C**) SO_2_, (**D**) NO_2_, (**E**) CO and (**F**) O_3_, respectively. The solid lines are odds ratios of acute glaucoma attack; the shaded areas were the 95% confidence intervals

The overall lag-response relationship curves in association of ambient air pollution exposure with outpatient visits for acute glaucoma on different lag day was showed in Fig. 2. The associated between air pollutants (PM_2.5_, PM_10_, NO_2_ and CO) and the odds of acute glaucoma visits was significant. The lag effect for PM_2.5_ and NO_2_ (lag0–3 days) was relatively longer for PM_10_ and CO (lag0–1). Specifically, an IQR increase in PM_2.5_ (26 μg/m^3^), PM_10_ (35 μg/m^3^), NO_2_ (27 μg/m^3^) and CO (0.5 mg/m^3^) was associated with 7% (OR: 1.07; 95%CI: 1.03–1.11), 3% (OR:1.03; 95% CI: 1.01–1.05), 12% (OR: 1.12; 95% CI: 1.08, 1.17) and 4% (OR: 1.04; 95% CI: 1.01, 1.07) higher odds of acute glaucoma visits (Table 2). Besides, the lag effect for SO_2_ and O_3_ associated acute glaucoma visits was not statistically significant.

**Table 2:** Odds ratios (95% confidence intervals) of acute glaucoma attack per IQR increase in ambient air pollution exposure, stratified by sex and age

Air pollution	Lag	Variables	Sub-groups	OR (95% CI)	*P* value	*P* value for interaction
PM_2.5_	0–3	Total		1.07 (1.03, 1.11)	< 0.001^*^	
		Sex	Male	1.06 (1.02, 1.11)	0.008^*^	0.722
			Female	1.07 (1.02, 1.13)	0.008^*^	
		Age (y)	18–44	1.05 (1.01, 1.09)	0.003^*^	0.245
			45–85	1.10 (1.03, 1.18)	0.012	
PM_10_	0–1	Total		1.03 (1.01, 1.05)	0.004^*^	
		Sex	Male	1.02 (0.98, 1.06)	0.331	0.491
			Female	1.03 (1.01, 1.07)	0.010	
		Age (y)	18–44	1.02 (0.99, 1.05)	0.166	0.461
			45–85	1.05 (1.02, 1.10)	0.014	
SO_2_	0–1	Total		1.00 (0.97, 1.04)	0.848	
		Sex	Male	1.00 (0.95, 1.06)	0.917	0.975
			Female	1.00 (0.96, 1.05)	0.878	
		Age (y)	18–44	0.99 (0.95, 1.04)	0.744	0.351
			45–85	1.03 (0.96, 1.10)	0.379	
NO_2_	0–3	Total		1.12 (1.08, 1.17)	< 0.001^*^	
		Sex	Male	1.10 (1.01, 1.20)	0.040	0.152
			Female	1.21 (1.12, 1.30)	< 0.001^*^	
		Age (y)	18–44	1.11 (0.99, 1.25)	0.115	0.240
			45–85	1.18 (1.10, 1.26)	< 0.001^*^	
CO	0–1	Total		1.04 (1.01, 1.07)	0.008^*^	
		Sex	Male	1.03 (0.99, 1.07)	0.198	0.360
			Female	1.06 (1.01, 1.11)	0.023	
		Age (y)	18–44	1.03 (0.99, 1.07)	0.117	0.278
			45–85	1.07 (1.01, 1.14)	0.019	
O_3_	0–1	Total		1.02 (0.98, 1.06)	0.319	
		Sex	Male	1.00 (0.94, 1.06)	0.953	0.349
			Female	1.03 (0.99, 1.08)	0.170	
		Age (y)	18–44	1.00 (0.95, 1.04)	0.875	0.104
			45–85	1.09 (1.01, 1.17)	0.023	

^*^Statistically significant estimate (*p* value < 0.0083)

**Fig. 3:**
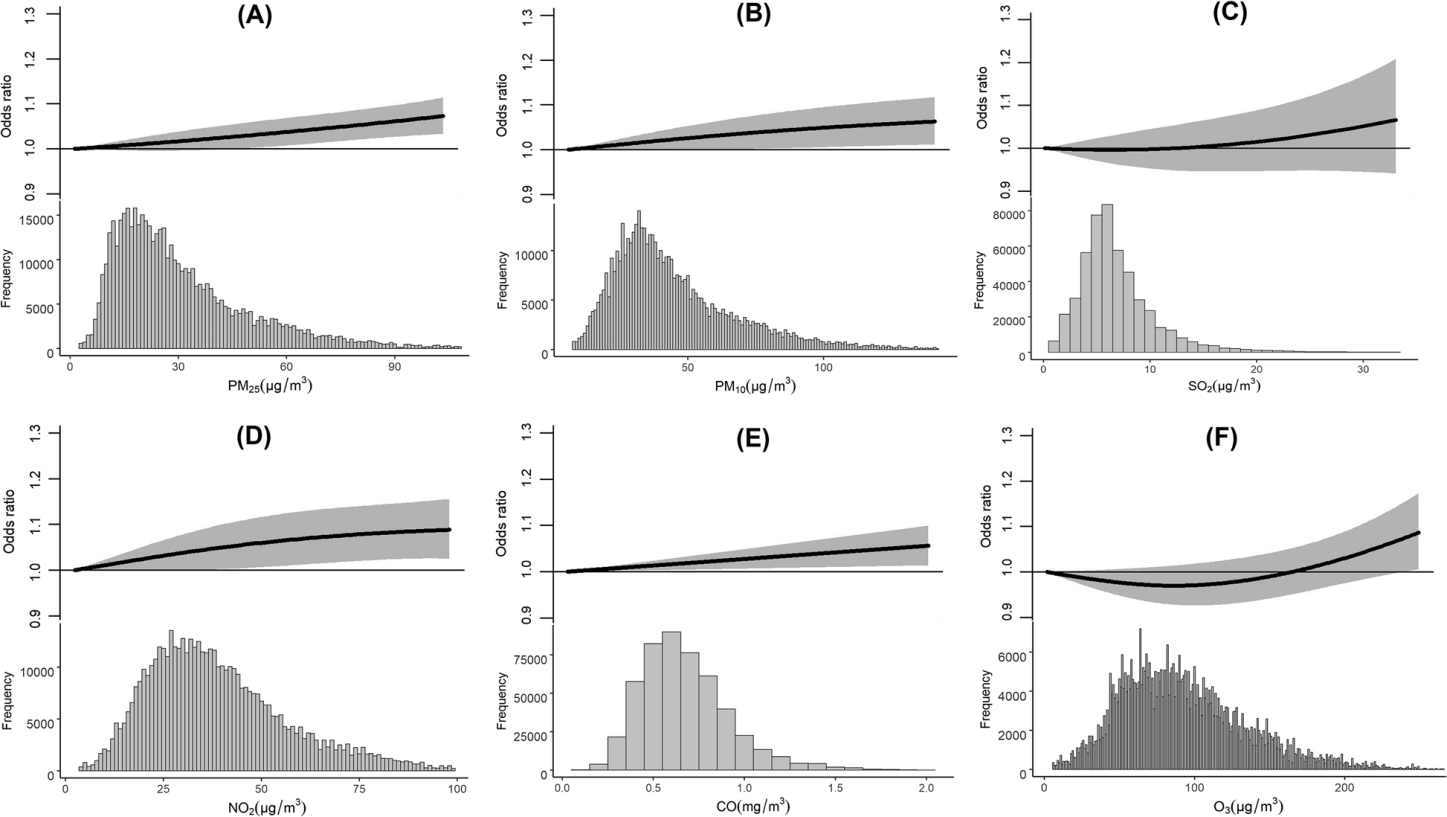
Cumulative exposure-response curves for associations between air pollution and acute glaucoma attack in Shanghai, China, January 2015 to March 2021. Panels A to F were the associations between OR of acute glaucoma attack and air pollution exposure, including (**A**) PM_2.5_, (**B**) PM_10_, (**C**) SO_2_, (**D**) NO_2_, (**E**) CO and (**F**) O_3_, respectively. These penalized splines regression models fit from the 0.1th to 99.9th percentiles of the concentrations of each pollutant, respectively. The solid lines are odds ratios of glaucoma; the shaded areas were the 95% confidence intervals

The associations between outpatient visits for acute glaucoma and air pollutants, including PM_2.5_, PM_10_, SO_2_, NO_2_, CO and O_3_, were ﻿illustrated in the cumulative E-R curves in Fig. 3. Lag days with significant effects (lag 0–3 days for PM_2.5_, NO_2_ and lag 0–1 days for PM_10_, SO_2_, CO and O_3_) was applied to plot the curves. In general, E-R curves for PM_2.5_, PM_10_ and CO were linear, but the acute glaucoma odds only significantly increased when the concentrations of PM_2.5_, and PM_10_ were above 50 μg/m^3^ and 60 μg/m^3^, respectively. The slope for NO_2_ exhibited significant increment over 40 μg/m^3^ and becomen flat over 80 μg/m^3^.

In addition, the stratified analysis showed suggestive effect modification of gender and age (Table 2). Female patients and the patients aged over 45 years were found to have relatively higher odds of glaucoma visits associated with air pollution, although no significant difference was found in difference analysis. And the result of in difference analysis was also unsignificant in terms of the association between acute glaucoma attack and other pollutants.

In the sensitive analysis, the associations between outpatient visits for acute glaucoma attack and air pollutants were relatively robust when one of the other five pollutants was adjusted in two-pollutants model (Fig. S1).

## Discussion

In this study, we observed an increase the risk of acute glaucoma associated with ambient air pollutants, with inconsistent delayed effects on extended lags. Exposure to air pollutants (PM_2.5_, PM_10_, SO_2_, NO_2_, and CO) was related to increased odds of outpatient visits for acute glaucoma. Specifically, PM_2.5_, PM_10_, NO_2_ and CO exhibited relatively stronger association on acute glaucoma outpatient visits with longer delayed effects.

PM_2.5_ was associated with glaucoma incidence across different countries and ethnic groups [17, 31, 33]. In UK, significant correlations were showed between PM_2.5_ exposure and occurrence of glaucoma [16, 17]. However, in the UK study, the glaucoma diagnosis was based on patient’s self-report without a clear diagnosis concerning the exact type of glaucoma, and the study conducted in Taiwan had a very small sample size of a few hundreds of primary angle closure glaucoma (PACG) patients [16]. In our current study, a big sample size of dataset were recruited over 14 thousand patients with clear diagnosis. Furthermore, both PM_2.5_ and PM_10_ showed a faster increment at higher concentrations (Fig. 3A).

It is possible that PM pollutions acted as a trigger for marked IOP elevation in angle closure glaucoma and glaucomatocyclitic crisis patients. Previous study showed that some PM particles could penetrate the cornea and entre the anterior chamber of the eye [35]. Topical administration of PM_2.5_ suspensions resulted in IOP elevation [35, 42], which was associated with increased oxidative stress and related NLRP3 inflammasome mediated pyroptosis in outflow control cells and tissues [35, 36]. In angle closure glaucoma there was an appositional or adhesion closure of the anterior chamber angle. It is possible that PM pollution may trigger angle closure by some mechnisms that previously existed in the narrow anterior chamber angle, thus is presented as an acute episode. In addition to anatomical predisposing factors, the PM in the anterior segment may cause oxidative stress and inflammation of the tissues contributing to the marked IOP elevation. Virus infections, such as infections of cytomegalovirus [43, 44], varicella-zoster virus [45], herpes simplex virus [45, 46] and *Helicobacter pylori* [47] and immune mediators [48] were found in the aqueous humor of glaucomatocyclitic crisis patients, which were thought as the initial events of the disease [24]. And it is known that the spread of virus was positively correlated to air pollutions [49] and air pollutions may cause immune disorder [50]. Thus, it is possible that PM particles could act as a carrier for the viruses and cause infections and inflammation, or by provoking immune response, which leads to IOP elevation in the human eye.

The ORs between PM_2.5_ or PM_10_ exposure and glaucoma outpatients incidence (PM_2.5_, OR:1.07; 95%CI: 1.03–1.11; PM_10_, OR:1.03; ﻿95% CI: 1.01–1.05; Table 2) were similar to the cohort study conducted in UK which reported that diagnosis of glaucoma was more likely to be reported by ﻿people in higher PM_2.5_ concentration areas (PM_2.5_ OR: 1.06; 95%CI:1.01–1.12) [17]. The discrepancy of the results may be attributed to specific study populations, geographic regions, sensitivities of glaucoma subtypes to PM_2.5_ exposure, differences of PM_2.5_ components and concentrations. These evidences suggest that air pollution may promote the initiation and progression of glaucoma.

NO_2_ and CO were major toxic atmospheric pollutants [51], however, their relationships with glaucoma incidence were scarcely studied. Based on our findings, gaseous pollutants, NO_2_ and CO, were mildly associated with odds of acute glaucoma attack, with the association occurred at lag 0–3 and 0–1 (Fig. 2). The invisible CO is a chemically-inert gas and inhaled CO can combine with hemoglobin to form carboxyhemoglobin, which makes hemoglobin lose the ability to carry oxygen [52]. NO_2_ is an ubiquitous atmospheric pollutant ﻿derived from emissions of NO, the major source of which are emissions from motor vehicles. Previous studies revealed that when SO_2_, NO_2_, and O_3_ ﻿increased 10 μg/m^3^ in and CO, 1 mg/m^3^, hospital admissions for ischemic stroke increased 1.37, 1.82, 0.01, and 3.24%, respectively [53]. NO_2_ inhalation exposure exerted injuries to lung, heart and brain, which were possibly related with oxidative stress and inflammation [54–57]. A time series prospective study conducted in Chiang Mai, Thailand reported that NO_2_ was positively associated with eye irritation (adjusted ORs (ROAORs: 1.024 to 1.229), and CO was positively related to lower heart and lung symptoms (adjusted ORs: 1.117 and 1.137) [58]. Large amount of CO could cause visual dysfunction [59]. It is interesting that low-dose CO inhalation protected RGCs from optic nerve injury [60], and carbon monoxide-releasing molecules (CORMs) derived from CO lowed IOP of rabbits in two ocular hypertension models [61]. Clearly these results were based on the effect of CO in a short term. This clinical population study suggestted that long term CO therapy for the treatment of glaucoma should be viewed with caution. The long-term effect and dose response of CO to the IOP is uncertain.

Female glaucoma patients seemed slightly more susceptible to air pollutants (Table 2), which is consistent with women having a higher odds of developing primary angle closure glaucoma [62–64]. Though the point estimates of the NO_2_ effect on acute glaucoma were more pronounced among the female and the patients aged over 65 years, no statistically significant differences was found in our current study. According to a meta-analysis, the estimated PACG prevalence in Chinese women was higher compared with men (1.9% vs 1.1%; adjusted OR: 1.75, 95% CI, 1.20–2.56; *P* = 0.004) [65]. In Korea, women had a 2.56 folds higher incidence rate of acute angle closure glaucoma than men [66]. Though a study reported female (56.6%) was more at odds of developing glaucomatocyclitic crisisris, most studies have shown a male predilection [67]. Additionally, although age was a strong risk factor of glaucoma [68, 69], the association between glaucoma incidence and air pollution was independent of age. It is possible that PM_2.5_ exposure might affect glaucoma patients at full life circle.

In different periods, air pollutants show different correlations: the principal components before the Spring Festival were O_3_ and NO_2_, and after the Spring Festival, they were PM_2.5_ and CO, while the principal components before the lockdown in 2020 were PM_2.5_ and CO, and during lockdown they were O_3_ and NO_2_ [70]. And the elements of PM_2.5_ also varied in different places, so are the toxicities of PM_2.5_. for eample, relatively high crustal elements such as Al, Si, Ti, and Fe were detected in PM_2.5_ which reflected the undergoing major construction nearby the campus in shanghai [71]. While air pollutants in Lanzhou are rich in polycyclic aromatic hydrocarbons (PAHs), which mainly comes from coal combustion industries [72]. Higher toxicity was observed in PAHs riched PM_2.5_ [73]. Hence, the different sources may cause the effect size differences, which needs further investigation.

There are several limitations to our study. First of all, the exposure level was based on the value of the nearest monitors matched to participants’ addresses and did not consider participants’ travel history. Secondly, besides ambient pollutions, mental state, diet, behavior and socioeconomic status may influence the outpatient visits as well. Thirdly, the outpatient data were collected from two hospitals in Shanghai whose residential addresses were in Shanghai, so more care needs to be taken in interpreting these results, and further investigation is needed to verify the association in a large scale, multi-centre study to control bias.

## Conclusions

Our study found that air pollution exposure was related to outpatient visits for glaucoma. And PM exposure showed stronger association with outpatient visits for acute glaucoma. This finding and the pathophysiology mechanisms need to be further confirmed and investigated.

## Supplementary Information

**Additional file 1: Fig. S1.** Flow chart of the inclusion and exclusion of study population. **Table S1.** Pearson correlation coefficient of air pollutants and meteorological variables. **Fig. S2.** Odds ratios (95% confidence intervals) of acute glaucoma attack per IQR increase in ambient air pollution exposure in two-pollutant model.

## Data Availability

The datasets generated and/or analysed during the current study are not publicly available, but are available from the corresponding author on reasonable request.
