# Supplementary material for: Acute exposure to air pollutants increase the risk of acute glaucoma
Source: BMC Public Health. 2022 Sep 20;22:1782. doi: 10.1186/s12889-022-14078-9 (PMC9487138; doi:10.1186/s12889-022-14078-9)
Supplement: Supplementary file 1 — Additional file 1: Fig. S1. Flow chart of the inclusion and exclusion of study population. Table S1. Pearson correlation coefficient of air pollutants and meteorological variables. Fig. S2. Odds ratios (95% confidence intervals) of acute glaucoma attack per IQR increase in ambient air pollution exposure in two-pollutant model. [file 12889_2022_14078_MOESM1_ESM.docx]

**Supplemental Materials**

**Acute exposure to air pollutants increase the risk of acute glaucoma**

#Liping Li^1,2^, #Yixiang Zhu^3^, Binze Han^1,2^, Renjie Chen^3, 5^, Xiaofei Man^6*^, Xinghuai Sun^1,2,4*^, Haidong Kan^3, 7, *^, Yuan Lei^1,2, *^

1. Department of Ophthalmology & Visual Science, Eye Institute, Eye & ENT Hospital, Shanghai Medical College, Fudan University, Shanghai 200031, China
2. NHC Key Laboratory of Myopia, Chinese Academy of Medical Sciences, and Shanghai Key Laboratory of Visual Impairment and Restoration (Fudan University), Shanghai 200031, China
3. School of Public Health, Key Lab of Public Health Safety of the Ministry of Education, NHC Key Lab of Health Technology Assessment, IRDR ICoE on Risk Interconnectivity and Governance on Weather/Climate Extremes Impact and Public Health, Fudan University, Shanghai, China
4. State Key Laboratory of Medical Neurobiology and MOE Frontiers Center for Brain Science, Institutes of Brain Science, Fudan University, Shanghai 200032, China
5. Shanghai Typhoon Institute/CMA, Shanghai Key Laboratory of Meteorology and Health, Shanghai, 200030, China
6. Department of Ophthalmology, Xinhua Hospital Affiliated to Shanghai Jiao Tong University School of Medicine, Shanghai, China, Shanghai, China
7. Children’s Hospital of Fudan University, National Center for Children’s Health, Shanghai, China

^*^Correspondence to Prof. Yuan Lei, [lilian0167@hotmail.com](mailto:lilian0167@hotmail.com), yuan_lei@fudan.edu.cn, Department of Ophthalmology & Visual Science, Eye Institute, Eye & ENT Hospital, Shanghai Medical College, Fudan University, Shanghai 200031, China; or Prof. Haidong Kan, School of Public Health, Fudan University, P.O. Box 249, 130 Dong-An Road, Shanghai 200032, China. E-mail address: [kanh@fudan.edu.cn](mailto:kanh@fudan.edu.cn). Xinghuai Sun, Department of Ophthalmology & Visual Science, Eye Institute, Eye & ENT Hospital, Shanghai Medical College, Fudan University, Shanghai, China. E-mail: xhsun@shmu.edu.cn. Xiaofei Man, manxiaofei@xinhuamed.com.cn

#These authors contributed equally to the work.

**Figure S1.** Flow chart of the inclusion and exclusion of study population

445,183 glaucoma patients registered in the database.

22,087 adult glaucoma patients with specific diagnosis were finally included

197,169 subjects with unknown characteristic information, or living out of the Shanghai, China were excluded.

225,487 cases of after glaucoma surgery, dispensing, prescribing, without specific sub-group diagnosis were excluded.

352 subjects, whose age were <18 or >85 were excluded.

247,926 glaucoma patients with complete characteristic information in Shanghai were included.

14,385 adult glaucoma patients diagnosed as acute glaucoma attack were finally included.

7,702 cases, who were diagnosed as open-closure glaucoma were excluded

**Table S1.** Pearson correlation coefficient of air pollutants and meteorological variables.

| Index | SO_2_ | NO_2_ | CO | O_3_ | PM_10_ | PM_2.5_ | Temperature | Humidity |
| --- | --- | --- | --- | --- | --- | --- | --- | --- |
| SO_2_ | 1 | 0.414^*^ | 0.349^*^ | 0.142^*^ | 0.461^*^ | 0.461^*^ | -0.221^*^ | -0.345^*^ |
| NO_2_ |  | 1 | 0.585^*^ | -0.157^*^ | 0.505^*^ | 0.658^*^ | -0.483^*^ | -0.146^*^ |
| CO |  |  | 1 | -0.059^*^ | 0.440^*^ | 0.671^*^ | -0.283^*^ | -0.032^*^ |
| O_3_ |  |  |  | 1 | 0.200^*^ | 0.101^*^ | 0.475^*^ | -0.311^*^ |
| PM_10_ |  |  |  |  | 1 | 0.672^*^ | -0.256^*^ | -0.385^*^ |
| PM_2.5_ |  |  |  |  |  | 1 | -0.349^*^ | -0.153^*^ |
| Temperature | |  |  |  |  |  | 1 | 0.273^*^ |
| Humidity | |  |  |  |  |  |  | 1 |

Note: ^*^P < 0.01


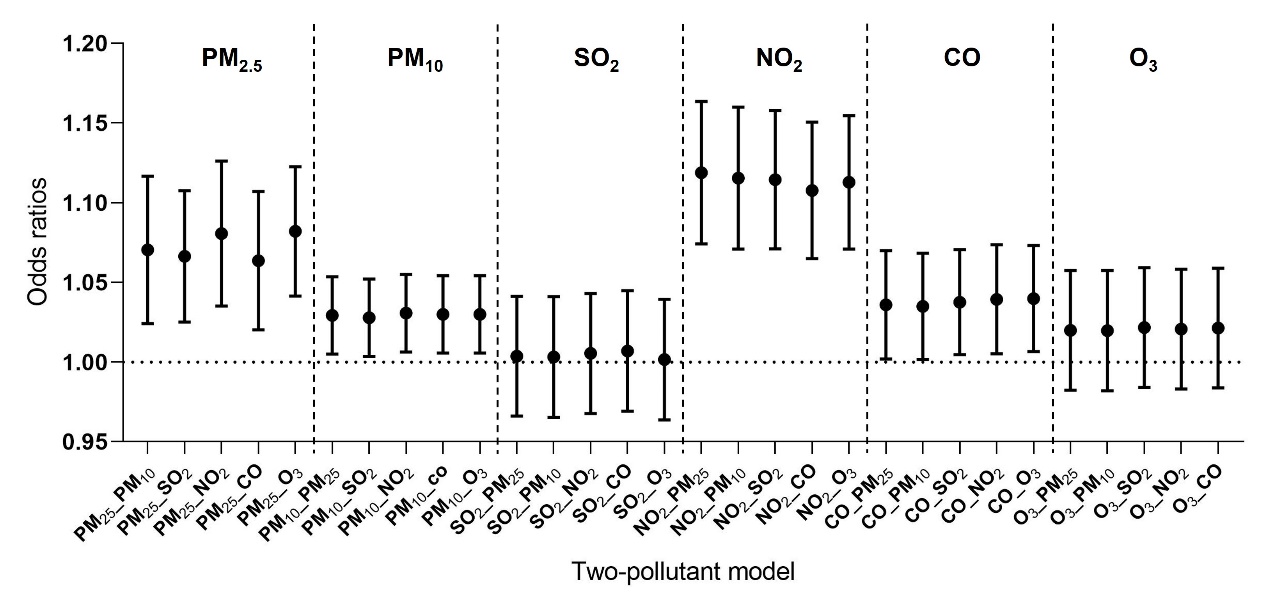


Figure S2. Odds ratios (95% confidence intervals) of acute glaucoma attack per IQR increase in ambient air pollution exposure in two-pollutant model.
